# Miscellany

**Published:** 1888-05

**Authors:** 


### Miscellany.

Inundation of the Lung.—An Italian surgeon, A. Riva, now proposes to cure tuberculosis by injecting antiseptic fluids into the pleural cavity, thus completely flooding the diseased lung. With a modified Mohr's tube and aspirating needle, an ounce of the antiseptic fluid selected is passed into the pleural sac. The injection should be made through the anterior part of the second intercostal space. Riva claims that there is little risk attending the operation, but mentions the following dangers:

1. Wound of a blood vessel and injection of the liquid in same.

2. Puncture and injection of bronchus or the pulmonary alveoli.

3. The toxic action from absorption of the antiseptic employed.—*Revista Medico-Quirurgica.*

Death of Dr. C. R. Agnew.—Dr. C. R. Agnew, the eminent ophthalmologist, died of acute peritonitis and typhlitis on the 18th inst.

Dr. Agnew was born in New York in 1830, and in 1852 graduated from the College of Physicians and Surgeons. For many years he was engaged in general practice. During the war he was very active in organizing the U. S. Sanitary Commission, and for three years a large portion of his time and talents were devoted to this benevolent work.

The Department of Ophthalmology and Otology in the College of Physicians and Surgeons, and the Manhattan Eye and Ear Hospital may be said to owe their foundation to his wise forethought, and it is largely due to his influence that ophthalmology has become the most highly developed of the surgical specialties in America.

"The Language of Medicine," by Dr. F. R. Campbell, has just been published, and may now be obtained of all booksellers. While the work was primarily designed for the instruction of medical students, we believe that every medical man who cares to use and pronounce correctly the technical terms of his profession, will find the book well worthy of his attention. That some such work as this, calling attention to the rules of English orthoepy, is needed, will be admitted by every practitioner. At a recent meeting of a gynecological society, for example, the word *gynecology* was pronounced in four different ways, when the rules of pronunciation admit of but one.

---

A "CONGRESS for the study of tuberculosis in man and the lower animals" will meet in Paris June 25 to 31, 1888. The committee in charge consist of Chauveau, president; Villemin, vice-president; and Batu, Le Blanc, Nocart, Rossignol, Cornil, Grancher, Lonnelogue and Verneuil, members of the committee.

---

"YOUR wife is in a very critical condition, and I think some specialist should be called in for consultation upon the case."

"There, now, doctor, I was right again. I told my wife long ago she ought to have proper medical treatment, but she thought you might be offended."—*Boston Journal of Health.*

---

SOME French physicians, Carrier, Rotellon and Baudin, claim that in the early stages of general paralysis, glycosuria is frequently present. Legrand du Saul insisted that it was very difficult to distinguish the early stages of general paresis from the nervous symptoms accompanying some forms of diabetes.

---

DR. B. has recently lost his beloved wife. "Doctor," said I, "you have my heartfelt sympathy; you must suffer grievously."

"Yes, but I took antipyrin according to the method Germain See recommends for hypochondriasis, and my grief was instantly relieved."—*Le Journal de Med. de Paris.*

---

LAWYER (in a hoarse whisper)—"Doctor, I've got such a cold this morning that I can't speak the truth." Doctor (sympathetically)—"I'm glad it isn't anything to interfere with your business."—*Boston Herald.*

CITIZEN—"In case of a sudden illness, doctor, what ought a person to do while waiting for a physician?"

Doctor—"Well, a physician's time is very valuable, you know, and the patient ought to get the two dollars ready, so that the doctor won't be bothered with making change."—*Scribner's*.

A WRITER in the *New England Medical Gazette* divides the medical world into homœopathists and allœopathists. We have, for a hundred years, been known as allopathists by the Hahnemannians, and do not understand the reasons for this change in spelling.

"PROFESSOR, what are your views concerning the schools of medicine and theology?"

Professor—"That depends upon circumstances. When I am slightly ill, I am a homœopathist and a Unitarian; but when I am very sick, I am an allopath and a Calvinist."

A LAW DISPENSARY has been established in New York, under the auspices of the People's Mission, for the benefit of the poor who require legal advice and cannot afford to pay for it.

MRS. DE BUFFINGTON says her husband suffered from suffusion into the plural, but the doctors drew off the water with an exasperator, and now he is incandescent.—*Weekly Medical Review*.

Hippolyte Brochin, for the past thirty-five years editor of *La Gazette des Hopitaux*, died in Paris, April 1st, aged eighty years.

